# Diacridinium *trans*-diaqua­bis(pyrazine-2,3-dicarboxyl­ato)cobaltate(II) hexa­hydrate

**DOI:** 10.1107/S1600536809053628

**Published:** 2009-12-19

**Authors:** Hossein Aghabozorg, Jafar Attar Gharamaleki, Mahdieh Parvizi, Zohreh Derikvand

**Affiliations:** a Faculty of Chemistry, Islamic Azad University, North Tehran Branch, Tehran, Iran; b Young Researchers Club, Islamic Azad University, North Tehran Branch, Tehran, Iran; c Department of Chemistry, Islamic Azad University, Khorramabad Branch, Khorramabad, Iran

## Abstract

The title compound, (C_13_H_10_N)_2_[Co(C_6_H_2_N_2_O_4_)_2_(H_2_O)_2_]·6H_2_O, consists of mononuclear *trans*-[Co(pz-2,3-dc)_2_(H_2_O)_2_]^2−^ complex anions, (acrH)^+^ cations and uncoordinated water mol­ecules (acr is acridine and pz-2,3-dcH_2_ is pyrazine-2,3-dicarboxylic acid). The Co^II^ atom, which lies on a crystallographic center of symmetry, has a slightly distorted octa­hedral coordination environment, with two N and two O atoms from the (pz-2,3-dc)^2−^ ligands in the equatorial plane and with two water mol­ecules in axial positions. In the crystal, the components are held together by two distinct N—H⋯O and C—H⋯O hydrogen bonds with *R*_2_^2^(8) graph-sets. The coordinated and uncoordinated water mol­ecules are also involved in O—H⋯O hydrogen bonds, which lead to the formation of layers with *R*_3_^3^(12) graph-set motifs. Extensive π–π stacking inter­actions between parallel aromatic rings of the acridinium ions, with distances ranging from 3.533 (1) to 3.613 (1) Å, occur in the structure.

## Related literature

For the crystal structure of pyrazine-2,3-dicarboxylic acid (pz-2,3-dcH_2_), see: Takusagawa & Shimada (1973 ▶). For complexes of (pz-2,3-dcH_2_) and manganese, copper, zinc, iron and cadmium, see: Zou *et al.* (1999 ▶); Konar *et al.* (2004 ▶); Li *et al.* (2003 ▶); Xu *et al.* (2008 ▶); Ma *et al.* (2006 ▶). For complexes of (pz-2,3-dcH_2_) with main group metals such as calcium, magnesium and sodium, see: Ptasiewicz-Bak & Leciejewicz (1997*a* ▶,*b* ▶); Tombul *et al.* (2006 ▶). For related structures of Co^II^ complexes with py-2,6-dcH_2_, see: Aghabozorg *et al.* (2007 ▶, 2009 ▶); Aghabozorg, Attar Gharamaleki *et al.* (2008 ▶). For a review article on proton-transfer agents and their metal complexes, see: Aghabozorg, Manteghi & Sheshmani (2008 ▶).
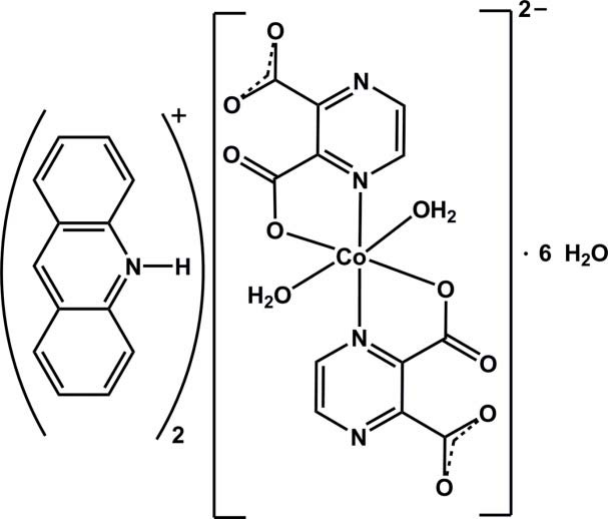


## Experimental

### 

#### Crystal data

(C_13_H_10_N)_2_[Co(C_6_H_2_N_2_O_4_)_2_(H_2_O)_2_]·6H_2_O
*M* *_r_* = 895.69
Triclinic, 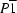

*a* = 6.9434 (15) Å
*b* = 9.682 (2) Å
*c* = 15.660 (5) Å
α = 94.60 (2)°
β = 98.59 (2)°
γ = 110.656 (16)°
*V* = 963.9 (4) Å^3^
*Z* = 1
Mo *K*α radiation
μ = 0.53 mm^−1^
*T* = 120 K
0.35 × 0.10 × 0.10 mm

#### Data collection

Bruker SMART 1000 diffractometer
Absorption correction: multi-scan (*SADABS*; Sheldrick, 1996 ▶) *T*_min_ = 0.828, *T*_max_ = 0.949
10681 measured reflections
5096 independent reflections
3880 reflections with *I* > 2σ(*I*)
*R*_int_ = 0.028

#### Refinement

*R*[*F* ^2^ > 2σ(*F* ^2^)] = 0.042
*wR*(*F* ^2^) = 0.101
*S* = 1.00
5096 reflections
277 parameters
H-atom parameters constrained
Δρ_max_ = 0.53 e Å^−3^
Δρ_min_ = −0.51 e Å^−3^

### 

Data collection: *SMART* (Bruker, 1998 ▶); cell refinement: *SAINT* (Bruker, 1998 ▶); data reduction: *SAINT*; program(s) used to solve structure: *SHELXTL* (Sheldrick, 2008 ▶); program(s) used to refine structure: *SHELXTL*; molecular graphics: *SHELXTL*; software used to prepare material for publication: *SHELXTL*.

## Supplementary Material

Crystal structure: contains datablocks I, global. DOI: 10.1107/S1600536809053628/om2301sup1.cif

Structure factors: contains datablocks I. DOI: 10.1107/S1600536809053628/om2301Isup2.hkl

Additional supplementary materials:  crystallographic information; 3D view; checkCIF report

## Figures and Tables

**Table 1:** Hydrogen-bond geometry (Å, °)

*D*—H⋯*A*	*D*—H	H⋯*A*	*D*⋯*A*	*D*—H⋯*A*
O1*W*—H1*WA*⋯O3*W*^i^	0.87	1.81	2.684 (2)	174
O1*W*—H1*WB*⋯O4*W*^ii^	0.90	1.77	2.658 (2)	174
O2*W*—H2*WA*⋯O2	0.90	1.92	2.813 (2)	172
O2*W*—H2*WB*⋯O4^iii^	0.88	1.90	2.776 (2)	177
O3*W*—H3*WA*⋯O1^iv^	0.88	1.95	2.806 (2)	165
O3*W*—H3*WB*⋯O2*W*^iv^	0.83	2.01	2.809 (2)	162
O4*W*—H4*WA*⋯O3	0.93	1.91	2.789 (2)	156
O4*W*—H4*WB*⋯N4^iii^	0.96	1.92	2.848 (2)	163
N9—H9⋯O4	0.92	1.74	2.648 (2)	167
C11—H11⋯O3	0.95	2.50	3.365 (3)	151
C12—H12⋯O1^v^	0.95	2.49	3.431 (3)	171
C16—H16⋯O2*W*^vi^	0.95	2.46	3.395 (3)	169

Symmetry codes: (i) 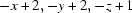; (ii) 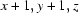; (iii) 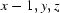; (iv) 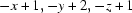; (v) 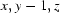; (vi) 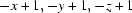.

**Fig. 1.**
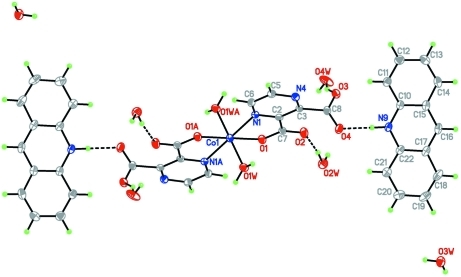
Molecular structure of (acrH)2[Co(pz-2,3-dc)2(H2O)2].6H2O; thermal ellipsoids are shown at the 50% probability level. Atoms marked with A suffix are related by the symmetry code: -x + 1, -y + 2, -z. The anionic complex lies on a crystallographic center of symmetry.

**Fig. 2.**
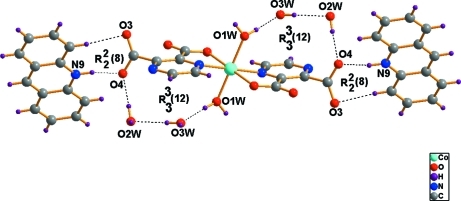
Hydrogen bonding with patterns of R22(8) and R33(12) graph sets link the different fragments together to form chains.

**Fig. 3.**
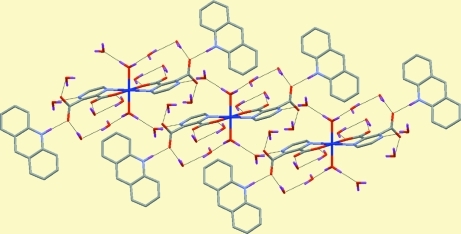
Crystal structure of (acrH)2[Co(pz-2,3-dc)2(H2O)2].6H2O compound; the spaces between two layers of (acrH)+ cations (as counter-ions) are filled with layers of [Co(pz-2,3-dc)2(H2O)2]2– fragments and uncoordinated water molecules.

**Fig. 4.**
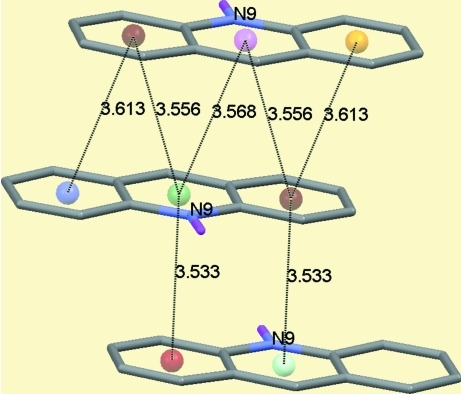
Extensive π-π stacking interaction between aromatic rings of acridinium ions, (acrH)+, with centroid-centroid distances ranging from 3.533 (1) to 3.613 (1) Å.

## supplementary crystallographic information

### Comment

Takusagawa & Shimada (1973) first determined the structure of pyrazine-2,3-dicarboxlic acid by single-crystal X-ray analysis. Pyrazine-2,3-dicarboxylic acid, (pz-2,3-dcH_2_), has proved to be well suited for the construction of multidimensional frameworks due to the presence of two adjacent carboxylate groups (O donor atoms) as substituents on the N-heterocyclic pyrazine ring (N donor atoms). A variety of metal-organic compounds of pyrazine-2,3-dicarboxylic acid have been characterized crystallographically. Among many reported compounds, complexes of transition metal ions, including manganese (Zou *et al.*, 1999), copper (Konar *et al.*,2004), zinc (Li *et al.*, 2003), iron (Xu *et al.*, 2008) and cadmium (Ma *et al.*, 2006), are extensively studied. Also, there are many reported compounds of (pz-2,3-dcH_2_) with main group metals such as calcium (Ptasiewicz-Bak & Leciejewicz, 1997*a*), magnesium (Ptasiewicz-Bak & Leciejewicz, 1997*b*) and sodium (Tombul *et al.*, 2006) complexes. In this paper, we report the synthesis and crystal structure of the title compound.

The reaction of pyrazine-2,3-dicarboxlic acid, acridine and cobalt(II) nitrate, resulted in the formation of (acrH)_2_[Co(py-2,3-dc)_2_(H_2_O)_2_]. 6H_2_O. This compound consists of an anionic complex, *trans*-[Co(pz-2,3-dc)_2_(H_2_O)_2_]^2–^, counter-ions, (acrH)^+^ and six uncoordinated water molecules. In the title compound, two carboxylic COOH protons have been transferred to non-coordinated pyridine rings of acridine. The central Co1 atom is six-coordinated by N1, O1, N1^i^ and O1^i^ atoms in the equatorial plane from two (pz-2,3-dc)^2–^ ligands and by two water molecules, O1W and O1W^i^, in the axial position [symmetry code (i), -*x* + 1,-*y* + 2, -*z*] (see Fig. 1). The water molecules are almost perpendicular to the plane of (pz-2,3-dc)^2–^ ligands, with O1W—Co1—N1, O1W—Co1—O1, O1W^i^—Co1—N1 and O1W^i^—Co1—O1 angles of 86.35 (6), 89.57 (6), 93.65 (6) and 90.43 (6)°, respectively.

The coordination environment around the Co^II^ may be considered as slightly distorted octahedral. The anionic complex lies on a crystallographic center of symmetry. The mean Co—N and Co—O bond distances are 2.1237 (15) and 2.0738 (14) Å, respectively, which are consistent with our previously reported data for Co^II^ complexes (Aghabozorg *et al.*, 2009; Aghabozorg, Attar Gharamaleki *et al.*, 2008; Aghabozorg, Manteghi, Sheshmani, 2008; Aghabozorg *et al.*, 2007).

In the structure of the title compound, (acrH)^+^ cations and [Co(pz-2,3-dc)_2_(H_2_O)_2_]^2–^ anions are linked together by two classical N9—H9···O4 and non-classical C11—H11···O3 hydrogen bonds by *R*_2_^2^(8) graph-set motifs. Also, the coordinated and uncoordinated water molecules are involved in O—H···O hydrogen bonds with O···O distances of 2.658 (2) to 2.813 (2) Å, which lead to the formation of chains with *R*_3_^3^(12) graph-set motifs (Fig. 2).

As can be seen from Fig. 3, in the crystal structure of (acrH)_2_[Co(pz-2,3-dc)_2_(H_2_O)_2_].6H_2_O, the spaces between two layers of (acrH)^+^ cations (as counter-ions) are filled with layers of [Co(pz-2,3-dc)_2_(H_2_O)_2_]^2–^ fragments and uncoordinated water molecules. Also an extensive π-π stacking interaction between aromatic rings of acridinium ions, (acrH)^+^, with centroid-centroid distances ranging from 3.533 (1) to 3.613 (1) Å are observed (Fig.4).

### Experimental

The reaction of cobalt(II) nitrate hexahydrate (72 mg, 0.25 mmol), acridine, acr, (90 mg, 0.5 mmol) and pyrazine-2,3-dicarboxylic acid, pz-2,3-dcH_2_, (84 mg, 0.5 mmol) in a 1:2:2 molar ratio in aqueous solution resulted in the formation of needle like, pale yellow, (acrH)_2_[Co(pz-2,3-dc)_2_(H_2_O)_2_]. 6H_2_O crystals.

### Refinement

The hydrogen atoms of NH groups and those of water molecules were found in difference Fourier synthesis. The H(C) atom positions were calculated. Hydrogen atoms were refined in isotropic approximation in riding model with the *U*_iso_(H) parameters equal to 1.2 *U*_eq_(Ci), for methyl groups equal to 1.5 *U*_eq_(Cii), where U(Ci) and U(Cii) are respectively the equivalent isotropic thermal parameters of the carbon atoms to which corresponding H atoms are bonded. H(N) and H(O) were refined using AFIX 3 starting from their difference map positions, with U_iso_ = 1.2 times the equivalent isotropic thermal parameter of the bonded atom.

### Crystal data

**Table:**

(C_13_H_10_N)_2_[Co(C_6_H_2_N_2_O_4_)_2_(H_2_O)_2_]·6H_2_O	*Z* = 1
*M**_r_* = 895.69	*F*(000) = 465
Triclinic, *P*1	*D*_x_ = 1.543 Mg m^−^^3^
Hall symbol: -P 1	Mo *K*α radiation, λ = 0.71073 Å
*a* = 6.9434 (15) Å	Cell parameters from 548 reflections
*b* = 9.682 (2) Å	θ = 3–30°
*c* = 15.660 (5) Å	µ = 0.53 mm^−^^1^
α = 94.60 (2)°	*T* = 120 K
β = 98.59 (2)°	Needles, yellow
γ = 110.656 (16)°	0.35 × 0.10 × 0.10 mm
*V* = 963.9 (4) Å^3^	

### Data collection

**Table:**

Bruker SMART 1000 diffractometer	5096 independent reflections
Radiation source: fine-focus sealed tube	3880 reflections with *I* > 2σ(*I*)
graphite	*R*_int_ = 0.028
φ and ω scans	θ_max_ = 29.0°, θ_min_ = 2.3°
Absorption correction: multi-scan (*SADABS*; Sheldrick, 1996)	*h* = −9→9
*T*_min_ = 0.828, *T*_max_ = 0.949	*k* = −13→13
10681 measured reflections	*l* = −21→21

### Refinement

**Table:**

Refinement on *F*^2^	Primary atom site location: structure-invariant direct methods
Least-squares matrix: full	Secondary atom site location: difference Fourier map
*R*[*F*^2^ > 2σ(*F*^2^)] = 0.042	Hydrogen site location: difference Fourier map
*wR*(*F*^2^) = 0.101	H-atom parameters constrained
*S* = 1.00	*w* = 1/[σ^2^(*F*_o_^2^) + (0.0367*P*)^2^ + 0.699*P*] where *P* = (*F*_o_^2^ + 2*F*_c_^2^)/3
5096 reflections	(Δ/σ)_max_ < 0.001
277 parameters	Δρ_max_ = 0.53 e Å^−^^3^
0 restraints	Δρ_min_ = −0.51 e Å^−^^3^

### Special details

**Table:**

Geometry. All e.s.d.'s (except the e.s.d. in the dihedral angle between two l.s. planes) are estimated using the full covariance matrix. The cell e.s.d.'s are taken into account individually in the estimation of e.s.d.'s in distances, angles and torsion angles; correlations between e.s.d.'s in cell parameters are only used when they are defined by crystal symmetry. An approximate (isotropic) treatment of cell e.s.d.'s is used for estimating e.s.d.'s involving l.s. planes.
Refinement. Refinement of *F*^2^ against ALL reflections. The weighted *R*-factor *wR* and goodness of fit *S* are based on *F*^2^, conventional *R*-factors *R* are based on *F*, with *F* set to zero for negative *F*^2^. The threshold expression of *F*^2^ > σ(*F*^2^) is used only for calculating *R*-factors(gt) *etc*. and is not relevant to the choice of reflections for refinement. *R*-factors based on *F*^2^ are statistically about twice as large as those based on *F*, and *R*- factors based on ALL data will be even larger.

### Fractional atomic coordinates and isotropic or equivalent isotropic displacement parameters (Å^2^)

**Table:**

	*x*	*y*	*z*	*U*_iso_*/*U*_eq_	
Co1	0.5000	1.0000	0.0000	0.01687 (10)	
O1W	0.7405 (2)	1.11233 (14)	0.10425 (8)	0.0206 (3)	
H1WA	0.8327	1.0726	0.1206	0.025*	
H1WB	0.7989	1.2121	0.1140	0.025*	
O1	0.3091 (2)	0.90307 (14)	0.08669 (8)	0.0183 (3)	
O2	0.2750 (2)	0.74313 (15)	0.18449 (9)	0.0234 (3)	
O3	0.3583 (2)	0.45588 (14)	0.18785 (9)	0.0209 (3)	
O4	0.6398 (2)	0.63005 (15)	0.27515 (8)	0.0228 (3)	
N1	0.5784 (2)	0.81013 (16)	0.01910 (10)	0.0164 (3)	
C2	0.5084 (3)	0.74911 (19)	0.08802 (11)	0.0153 (3)	
C3	0.5842 (3)	0.64696 (19)	0.12446 (11)	0.0155 (3)	
N4	0.7276 (2)	0.60676 (17)	0.09142 (10)	0.0184 (3)	
C5	0.7905 (3)	0.6652 (2)	0.02152 (12)	0.0190 (4)	
H5	0.8889	0.6364	−0.0040	0.023*	
C6	0.7162 (3)	0.7669 (2)	−0.01489 (12)	0.0184 (4)	
H6	0.7641	0.8063	−0.0648	0.022*	
C7	0.3499 (3)	0.8006 (2)	0.12333 (11)	0.0164 (4)	
C8	0.5162 (3)	0.5715 (2)	0.20244 (12)	0.0176 (4)	
N9	0.6756 (2)	0.47469 (17)	0.40425 (10)	0.0178 (3)	
H9	0.6439	0.5260	0.3606	0.021*	
C10	0.6512 (3)	0.3295 (2)	0.38850 (12)	0.0180 (4)	
C11	0.5757 (3)	0.2510 (2)	0.30266 (12)	0.0219 (4)	
H11	0.5427	0.2997	0.2555	0.026*	
C12	0.5512 (3)	0.1052 (2)	0.28880 (13)	0.0254 (4)	
H12	0.4993	0.0523	0.2313	0.031*	
C13	0.6007 (3)	0.0294 (2)	0.35744 (14)	0.0270 (4)	
H13	0.5827	−0.0726	0.3456	0.032*	
C14	0.6742 (3)	0.1031 (2)	0.44064 (14)	0.0244 (4)	
H14	0.7078	0.0521	0.4865	0.029*	
C15	0.7007 (3)	0.2556 (2)	0.45909 (12)	0.0196 (4)	
C16	0.7708 (3)	0.3361 (2)	0.54300 (12)	0.0216 (4)	
H16	0.8046	0.2886	0.5907	0.026*	
C17	0.7918 (3)	0.4849 (2)	0.55801 (12)	0.0209 (4)	
C18	0.8584 (3)	0.5710 (3)	0.64269 (13)	0.0262 (4)	
H18	0.8900	0.5263	0.6921	0.031*	
C19	0.8771 (3)	0.7159 (3)	0.65353 (13)	0.0289 (5)	
H19	0.9211	0.7717	0.7103	0.035*	
C20	0.8314 (3)	0.7843 (2)	0.58062 (14)	0.0265 (4)	
H20	0.8468	0.8862	0.5893	0.032*	
C21	0.7655 (3)	0.7068 (2)	0.49774 (13)	0.0222 (4)	
H21	0.7351	0.7541	0.4494	0.027*	
C22	0.7435 (3)	0.5551 (2)	0.48544 (12)	0.0182 (4)	
O2W	0.0497 (2)	0.83209 (16)	0.29844 (9)	0.0253 (3)	
H2WA	0.1136	0.7940	0.2619	0.030*	
H2WB	−0.0814	0.7706	0.2906	0.030*	
O3W	0.9656 (2)	0.99532 (15)	0.83648 (9)	0.0249 (3)	
H3WA	0.8947	1.0289	0.8693	0.030*	
H3WB	0.9717	1.0336	0.7911	0.030*	
O4W	−0.0631 (2)	0.40692 (16)	0.12903 (11)	0.0354 (4)	
H4WA	0.0802	0.4505	0.1498	0.042*	
H4WB	−0.1329	0.4762	0.1288	0.042*	

### Atomic displacement parameters (Å^2^)

**Table:**

	*U*^11^	*U*^22^	*U*^33^	*U*^12^	*U*^13^	*U*^23^
Co1	0.01858 (19)	0.01624 (18)	0.01689 (18)	0.00754 (14)	0.00289 (14)	0.00433 (13)
O1W	0.0203 (7)	0.0165 (6)	0.0235 (7)	0.0078 (5)	−0.0020 (5)	0.0013 (5)
O1	0.0197 (7)	0.0186 (6)	0.0206 (7)	0.0107 (5)	0.0053 (5)	0.0055 (5)
O2	0.0269 (7)	0.0250 (7)	0.0257 (7)	0.0136 (6)	0.0131 (6)	0.0110 (6)
O3	0.0201 (7)	0.0187 (7)	0.0229 (7)	0.0056 (5)	0.0034 (5)	0.0059 (5)
O4	0.0231 (7)	0.0261 (7)	0.0162 (6)	0.0059 (6)	0.0014 (5)	0.0056 (5)
N1	0.0192 (8)	0.0143 (7)	0.0147 (7)	0.0058 (6)	0.0022 (6)	0.0014 (6)
C2	0.0153 (8)	0.0130 (8)	0.0158 (8)	0.0048 (7)	0.0003 (7)	−0.0004 (6)
C3	0.0170 (8)	0.0138 (8)	0.0145 (8)	0.0051 (7)	0.0009 (7)	0.0010 (6)
N4	0.0200 (8)	0.0174 (8)	0.0190 (8)	0.0083 (6)	0.0035 (6)	0.0034 (6)
C5	0.0209 (9)	0.0189 (9)	0.0187 (9)	0.0088 (8)	0.0051 (7)	0.0008 (7)
C6	0.0210 (9)	0.0187 (9)	0.0170 (9)	0.0083 (7)	0.0051 (7)	0.0040 (7)
C7	0.0161 (9)	0.0155 (8)	0.0167 (8)	0.0053 (7)	0.0018 (7)	0.0019 (7)
C8	0.0196 (9)	0.0191 (9)	0.0184 (9)	0.0112 (7)	0.0048 (7)	0.0059 (7)
N9	0.0164 (7)	0.0229 (8)	0.0145 (7)	0.0076 (6)	0.0024 (6)	0.0049 (6)
C10	0.0140 (8)	0.0232 (9)	0.0172 (9)	0.0066 (7)	0.0035 (7)	0.0055 (7)
C11	0.0197 (9)	0.0261 (10)	0.0186 (9)	0.0075 (8)	0.0021 (7)	0.0038 (8)
C12	0.0225 (10)	0.0259 (10)	0.0235 (10)	0.0049 (8)	0.0031 (8)	0.0005 (8)
C13	0.0239 (10)	0.0197 (10)	0.0358 (12)	0.0060 (8)	0.0055 (9)	0.0045 (8)
C14	0.0207 (10)	0.0260 (10)	0.0292 (10)	0.0095 (8)	0.0057 (8)	0.0125 (8)
C15	0.0140 (8)	0.0267 (10)	0.0198 (9)	0.0083 (8)	0.0045 (7)	0.0076 (8)
C16	0.0160 (9)	0.0334 (11)	0.0187 (9)	0.0107 (8)	0.0054 (7)	0.0113 (8)
C17	0.0152 (9)	0.0336 (11)	0.0155 (9)	0.0103 (8)	0.0036 (7)	0.0052 (8)
C18	0.0193 (10)	0.0457 (13)	0.0158 (9)	0.0152 (9)	0.0028 (7)	0.0025 (9)
C19	0.0205 (10)	0.0442 (13)	0.0200 (10)	0.0124 (9)	0.0025 (8)	−0.0059 (9)
C20	0.0189 (10)	0.0321 (11)	0.0286 (11)	0.0111 (9)	0.0052 (8)	−0.0031 (9)
C21	0.0187 (9)	0.0275 (10)	0.0222 (9)	0.0108 (8)	0.0044 (7)	0.0032 (8)
C22	0.0125 (8)	0.0265 (10)	0.0157 (8)	0.0072 (7)	0.0039 (7)	0.0025 (7)
O2W	0.0227 (7)	0.0293 (8)	0.0244 (7)	0.0093 (6)	0.0071 (6)	0.0042 (6)
O3W	0.0291 (8)	0.0299 (8)	0.0247 (7)	0.0196 (6)	0.0079 (6)	0.0088 (6)
O4W	0.0200 (7)	0.0181 (7)	0.0642 (11)	0.0077 (6)	−0.0030 (7)	0.0021 (7)

### Geometric parameters (Å, °)

**Table:**

Co1—O1W	2.0631 (14)	C11—C12	1.356 (3)
Co1—O1W^i^	2.0631 (14)	C11—H11	0.9500
Co1—O1^i^	2.0846 (14)	C12—C13	1.416 (3)
Co1—O1	2.0846 (14)	C12—H12	0.9500
Co1—N1	2.1237 (15)	C13—C14	1.365 (3)
Co1—N1^i^	2.1237 (15)	C13—H13	0.9500
O1W—H1WA	0.8741	C14—C15	1.422 (3)
O1W—H1WB	0.8956	C14—H14	0.9500
O1—C7	1.279 (2)	C15—C16	1.396 (3)
O2—C7	1.236 (2)	C16—C17	1.393 (3)
O3—C8	1.235 (2)	C16—H16	0.9500
O4—C8	1.271 (2)	C17—C18	1.426 (3)
N1—C6	1.332 (2)	C17—C22	1.427 (3)
N1—C2	1.344 (2)	C18—C19	1.357 (3)
C2—C3	1.396 (2)	C18—H18	0.9500
C2—C7	1.513 (2)	C19—C20	1.416 (3)
C3—N4	1.344 (2)	C19—H19	0.9500
C3—C8	1.523 (2)	C20—C21	1.372 (3)
N4—C5	1.333 (2)	C20—H20	0.9500
C5—C6	1.386 (3)	C21—C22	1.416 (3)
C5—H5	0.9500	C21—H21	0.9500
C6—H6	0.9500	O2W—H2WA	0.9045
N9—C10	1.352 (2)	O2W—H2WB	0.8778
N9—C22	1.359 (2)	O3W—H3WA	0.8802
N9—H9	0.9214	O3W—H3WB	0.8268
C10—C11	1.416 (3)	O4W—H4WA	0.9266
C10—C15	1.428 (3)	O4W—H4WB	0.9560
			
O1W—Co1—O1W^i^	180.00 (8)	C22—N9—H9	114.8
O1W—Co1—O1^i^	90.43 (6)	N9—C10—C11	120.24 (16)
O1W^i^—Co1—O1^i^	89.57 (6)	N9—C10—C15	119.61 (17)
O1W—Co1—O1	89.57 (6)	C11—C10—C15	120.15 (18)
O1W^i^—Co1—O1	90.43 (6)	C12—C11—C10	119.03 (18)
O1^i^—Co1—O1	180.0	C12—C11—H11	120.5
O1W—Co1—N1	86.35 (6)	C10—C11—H11	120.5
O1W^i^—Co1—N1	93.65 (6)	C11—C12—C13	122.01 (19)
O1^i^—Co1—N1	101.76 (6)	C11—C12—H12	119.0
O1—Co1—N1	78.24 (6)	C13—C12—H12	119.0
O1W—Co1—N1^i^	93.65 (6)	C14—C13—C12	119.96 (19)
O1W^i^—Co1—N1^i^	86.35 (6)	C14—C13—H13	120.0
O1^i^—Co1—N1^i^	78.24 (6)	C12—C13—H13	120.0
O1—Co1—N1^i^	101.76 (6)	C13—C14—C15	120.42 (18)
N1—Co1—N1^i^	180.000 (1)	C13—C14—H14	119.8
Co1—O1W—H1WA	118.4	C15—C14—H14	119.8
Co1—O1W—H1WB	121.9	C16—C15—C14	123.17 (18)
H1WA—O1W—H1WB	111.1	C16—C15—C10	118.39 (18)
C7—O1—Co1	116.07 (11)	C14—C15—C10	118.43 (18)
C6—N1—C2	118.22 (15)	C17—C16—C15	121.10 (17)
C6—N1—Co1	128.58 (12)	C17—C16—H16	119.5
C2—N1—Co1	111.60 (12)	C15—C16—H16	119.5
N1—C2—C3	120.57 (16)	C16—C17—C18	123.23 (18)
N1—C2—C7	116.06 (15)	C16—C17—C22	118.67 (17)
C3—C2—C7	123.36 (16)	C18—C17—C22	118.11 (19)
N4—C3—C2	120.99 (16)	C19—C18—C17	120.76 (19)
N4—C3—C8	114.13 (15)	C19—C18—H18	119.6
C2—C3—C8	124.87 (16)	C17—C18—H18	119.6
C5—N4—C3	117.53 (15)	C18—C19—C20	120.36 (19)
N4—C5—C6	121.74 (17)	C18—C19—H19	119.8
N4—C5—H5	119.1	C20—C19—H19	119.8
C6—C5—H5	119.1	C21—C20—C19	121.5 (2)
N1—C6—C5	120.89 (17)	C21—C20—H20	119.3
N1—C6—H6	119.6	C19—C20—H20	119.3
C5—C6—H6	119.6	C20—C21—C22	118.87 (19)
O2—C7—O1	126.20 (17)	C20—C21—H21	120.6
O2—C7—C2	118.32 (16)	C22—C21—H21	120.6
O1—C7—C2	115.47 (15)	N9—C22—C21	120.25 (17)
O3—C8—O4	127.19 (17)	N9—C22—C17	119.30 (18)
O3—C8—C3	117.46 (16)	C21—C22—C17	120.45 (17)
O4—C8—C3	115.12 (16)	H2WA—O2W—H2WB	107.6
C10—N9—C22	122.92 (16)	H3WA—O3W—H3WB	110.8
C10—N9—H9	122.3	H4WA—O4W—H4WB	113.8
			
O1W—Co1—O1—C7	−73.69 (13)	C2—C3—C8—O3	86.2 (2)
O1W^i^—Co1—O1—C7	106.31 (13)	N4—C3—C8—O4	81.2 (2)
N1—Co1—O1—C7	12.67 (12)	C2—C3—C8—O4	−99.0 (2)
N1^i^—Co1—O1—C7	−167.33 (12)	C22—N9—C10—C11	178.69 (17)
O1W—Co1—N1—C6	−88.80 (16)	C22—N9—C10—C15	−0.9 (3)
O1W^i^—Co1—N1—C6	91.19 (16)	N9—C10—C11—C12	−179.51 (18)
O1^i^—Co1—N1—C6	0.87 (16)	C15—C10—C11—C12	0.1 (3)
O1—Co1—N1—C6	−179.13 (16)	C10—C11—C12—C13	−0.6 (3)
O1W—Co1—N1—C2	76.28 (12)	C11—C12—C13—C14	0.4 (3)
O1W^i^—Co1—N1—C2	−103.72 (12)	C12—C13—C14—C15	0.3 (3)
O1^i^—Co1—N1—C2	165.96 (12)	C13—C14—C15—C16	178.53 (19)
O1—Co1—N1—C2	−14.04 (12)	C13—C14—C15—C10	−0.7 (3)
C6—N1—C2—C3	2.1 (3)	N9—C10—C15—C16	0.9 (3)
Co1—N1—C2—C3	−164.70 (13)	C11—C10—C15—C16	−178.74 (17)
C6—N1—C2—C7	−179.20 (15)	N9—C10—C15—C14	−179.83 (17)
Co1—N1—C2—C7	14.00 (19)	C11—C10—C15—C14	0.6 (3)
N1—C2—C3—N4	−0.3 (3)	C14—C15—C16—C17	−179.19 (18)
C7—C2—C3—N4	−178.91 (16)	C10—C15—C16—C17	0.1 (3)
N1—C2—C3—C8	179.89 (16)	C15—C16—C17—C18	178.62 (18)
C7—C2—C3—C8	1.3 (3)	C15—C16—C17—C22	−1.0 (3)
C2—C3—N4—C5	−1.6 (3)	C16—C17—C18—C19	179.72 (19)
C8—C3—N4—C5	178.23 (16)	C22—C17—C18—C19	−0.7 (3)
C3—N4—C5—C6	1.7 (3)	C17—C18—C19—C20	−0.3 (3)
C2—N1—C6—C5	−2.0 (3)	C18—C19—C20—C21	0.7 (3)
Co1—N1—C6—C5	162.24 (14)	C19—C20—C21—C22	−0.1 (3)
N4—C5—C6—N1	0.1 (3)	C10—N9—C22—C21	−179.90 (17)
Co1—O1—C7—O2	169.89 (15)	C10—N9—C22—C17	0.0 (3)
Co1—O1—C7—C2	−8.93 (19)	C20—C21—C22—N9	179.06 (17)
N1—C2—C7—O2	177.20 (16)	C20—C21—C22—C17	−0.8 (3)
C3—C2—C7—O2	−4.1 (3)	C16—C17—C22—N9	1.0 (3)
N1—C2—C7—O1	−3.9 (2)	C18—C17—C22—N9	−178.67 (16)
C3—C2—C7—O1	174.79 (16)	C16—C17—C22—C21	−179.16 (17)
N4—C3—C8—O3	−93.7 (2)	C18—C17—C22—C21	1.2 (3)

Symmetry codes: (i) −*x*+1, −*y*+2, −*z*.

### Hydrogen-bond geometry (Å, °)

**Table:**

*D*—H···*A*	*D*—H	H···*A*	*D*···*A*	*D*—H···*A*
O1W—H1WA···O3W^ii^	0.87	1.81	2.684 (2)	174
O1W—H1WB···O4W^iii^	0.90	1.77	2.658 (2)	174
O2W—H2WA···O2	0.90	1.92	2.813 (2)	172
O2W—H2WB···O4^iv^	0.88	1.90	2.776 (2)	177
O3W—H3WA···O1^v^	0.88	1.95	2.806 (2)	165
O3W—H3WB···O2W^v^	0.83	2.01	2.809 (2)	162
O4W—H4WA···O3	0.93	1.91	2.789 (2)	156
O4W—H4WB···N4^iv^	0.96	1.92	2.848 (2)	163
N9—H9···O4	0.92	1.74	2.648 (2)	167
C11—H11···O3	0.95	2.50	3.365 (3)	151
C12—H12···O1^vi^	0.95	2.49	3.431 (3)	171
C16—H16···O2W^vii^	0.95	2.46	3.395 (3)	169

Symmetry codes: (ii) −*x*+2, −*y*+2, −*z*+1; (iii) *x*+1, *y*+1, *z*;  (iv) *x*−1, *y*, *z*; (v) −*x*+1, −*y*+2, −*z*+1; (vi) *x*, *y*−1, *z*; (vii) −*x*+1, −*y*+1, −*z*+1.
